# *Sargassum*@magnetite Composite EDTA-Functionalized for the Potential Removal of Mercury

**DOI:** 10.3390/polym15061405

**Published:** 2023-03-11

**Authors:** Diana Issell Sandoval-Cárdenas, Hector Pool, Sarai E. Favela-Camacho, José Santos-Cruz, Juan Campos-Guillén, Miguel Angel Ramos-López, Eloy Rodríguez-deLeón, Jessica Viridiana Urbina-Arroyo, Aldo Amaro-Reyes

**Affiliations:** 1 Facultad de Química, Universidad Autónoma de Querétaro, Cerro de las Campanas, Santiago de Querétaro 76010, Mexico; 2 Graduate and Research Division, Engineering Faculty, Universidad Autónoma de Querétaro, Cerro de las Campanas, Santiago de Querétaro 76010, Mexico; 3 Instituto de Ingeniería y Tecnología, Universidad Autónoma de Ciudad Juárez, Avenida del Charro s/n y, Calle Henry Dunant, Omega, Ciudad Juárez 32584, Mexico; 4 DIPA, Facultad de Química, Universidad Autónoma de Querétaro, Cerro de las Campanas, Santiago de Querétaro 76010, Mexico

**Keywords:** alkaline co-precipitation, *Sargassum* spp., magnetic composite, heavy metal adsorption, mercury removal

## Abstract

*Sargassum* spp. affects the Caribbean shores; thus, its remotion or valorization is a priority. This work aimed to synthesize a low-cost magnetically retrievable Hg^+2^ adsorbent functionalized with ethylenediaminetetraacetic acid (EDTA) based on *Sargassum*. The *Sargassum* was solubilized to synthesize by co-precipitation a magnetic composite. A central composite design was assessed to maximize the adsorption of Hg^+2^. The solids yield magnetically attracted mass, and the saturation magnetizations of the functionalized composite were 60.1 ± 17.2%, 75.9 ± 6.6%, and 1.4 emu g^−1^. The functionalized magnetic composite yielded 29.8 ± 0.75 mg Hg^+2^ g^−1^ of chemisorption after 12 h, pH 5, and 25 °C achieving 75% Hg^+2^ adsorption after four reuse cycles. Crosslinking and functionalization with Fe_3_O_4_ and EDTA created differences in surface roughness as well as the thermal events of the composites. The Fe_3_O_4_@*Sargassum*@EDTA composite was a magnetically recovered biosorbent of Hg^2+^.

## 1. Introduction

Mercury (Hg^2+^) is increasingly entering the environment through human activities, such as mining, chlorine, energy and fuel production, and electronic and chemical industries, discharging it into wastewater [1,2]. Mercury is the second most toxic heavy metal, all its forms have toxicity, and therefore this pollutant causes environmental concerns [2,3]. The most toxic form of mercury is in the form of salts converted by algae and bacteria in water into the neurotoxic substance methylmercury, which bioaccumulates through the aquatic food chain, with it being a health risk to humans and wildlife [4,5]. The maximum permissible limits of mercury in drinking water are around 0.002 mg L^−1^ [2,5], while wastewater limits in Mexico are around 0.01 and 0.005 mg L^−1^ [6]; however, elevated concentrations in the range of 0.01–2.5 mg L^−1^ are reported in contaminated waters worldwide [7]. Due to its high toxicity, retrieving mercury from water is vital.

Hg^2+^ removal from wastewater is mainly carried out through precipitation/coprecipitation, as it is a robust method towards water hardness and the presence of other contaminants; however, it is expensive and often coupled with adsorption as a polishing technology. Membrane filtration is expensive, sensitive to other contaminants, and produces a large volume of residues, while other methods such as ion exchange, reverse osmosis, complexation/sequestration, and electrochemical operations are commercially impractical, inefficient, expensive, and yield toxic intermediate products [1,8]. Currently, adsorption is considered a cost-effective technology for the treatment of heavy metals in wastewater that has several advantages over conventional methods due to high efficiency, low cost, ease of operation, the possibility of metal recovery, and adsorbent regeneration as well as no secondary pollution. The most common and versatile adsorbent is activated carbon; however, it is expensive [9]. Other high-availability sustainable biomass adsorbents include land [10] and aquatic plants [11]. Biosorbents, such as algae, are eco-friendly and have shown higher heavy metal removal from wastewater, less sludge production, a high adsorption-to-volume ratio, and economic effectiveness [2]. *Sargassum* spp. is an abundant invasive brown seaweed that has besieged Caribbean coastlines, negatively impacting marine ecosystems and economic activities such as tourism and fishing, so there is an urgent need for its utilization as a possible beneficial raw material [12,13]. *Sargassum* spp. is projected to be an adequate biosorbent due to its high metal-ion-recovering capacity, abundance, and acceptable properties supporting cultivation for industrial needs [3,14]. Mercury adsorption has been scarcely evaluated [2]; in algal biomass adsorption, it is due to a chemisorption process where the covalent bonding between the carboxyl groups creates sites for metal ion sharing or exchange, overlapping, and complexation of polysaccharides and siloxane [2,15]. 

However, raw biomass should be prepared with standard techniques, such as washing, drying, or grinding, combined with chemical activation to increase the adsorption of metal ions through electrostatic interaction [16]. Furthermore, the extraction process of fucoidan, laminarin, and alginate contained in *Sargassum* spp. [17] includes several complex and time-consuming steps, and the correct adjustment of extraction parameters (e.g., time, temperature, and solvent) dramatically influences the yield, physical, chemical, and biochemical properties [18]. 

Additionally, chemical modification of natural adsorbents with ethylenediaminetetraacetic acid (EDTA) introduces desired functional groups for heavy metal ions removal [19]. On the other hand, magnetically recyclable bifunctional adsorbents facilitate their recovery using a permanent magnet, therefore avoiding secondary pollution [20]. Although some articles have already discussed heavy metal removal by adsorbents derived from low-cost materials, the current trend development of adsorbents made of algae and seaweed for heavy metal removal from water is still rare [16]. Thus, natural adsorbents with minimal chemical treatment, relatively low cost, availability, and reusability could be alternatives for the removal of heavy metals in wastewater. Hence, the objective of the present work was to investigate the synthesis of a magnetic composite of *Sargassum* spp. functionalized with EDTA as a potential adsorbent material for aqueous Hg^+2^ removal.

## 2. Materials and Methods

### 2.1. Materials

*Sargassum* spp. was collected in July 2020 from three sites belonging to the Caribbean coasts of Quintana Roo, Mexico (20°12′2.072″ N, 87°26′1.88″ W, 20°37′18.188″ N, 87°4′26.982″ O, 20°51′12.936″ N, 86°52′18.948″ W), according to what was reported by [13]. The samples were transported in low-density polyethylene bags inside an expanded polystyrene container at room temperature and were then washed thoroughly with potable water and five times with deionized water to remove the residual sand. After, the macroalgae were dried for 12 h at 50 °C and ground to 420 µm particle sizes. All chemicals were analytical grade and acquired from Merck KGaA, Darmstadt, Germany.

### 2.2. Solubilization of Sargassum spp. Polysaccharides

The effect of the acidic or alkaline medium and temperature on the solubilization of *Sargassum* spp. polysaccharides were evaluated using solutions of NaOH, Na_2_CO_3_, HCl, and CH_3_COOH at concentrations of 0.5, 1.5, and 2.5% p v^−1^ and heated to 60 or 80 °C while kept under constant stirring for 2 h at 200 rpm [21]. Subsequently, the algae suspensions were vacuum filtered using Whatman No. 4 filter paper and the solids retained on the paper were dried at 60 °C for 18 h and weighed to determine the percentage of solubility. The filtrate obtained was stored at 4 °C until use. The percentage of solubility was calculated according to Equation (1):

(1) $$\mathrm{solubility}\;\left(\%\right)=\frac{\left(\mathrm{initial}\;\mathrm{weight}\;\mathrm{of}\;Sargassum\;\mathrm{spp}.-\mathrm{retained}\;\mathrm{weight}\;\mathrm{in}\;\mathrm{filter}\;\mathrm{paper}\right)}{\mathrm{initial}\;\mathrm{weight}\;\mathrm{of}\;Sargassum\;\mathrm{spp}.}\times 100$$

### 2.3. Synthesis of the Magnetic Sargassum Composite (Fe_3_O_4_@Sargassum) Functionalized with EDTA (Fe_3_O_4_@Sargassum@EDTA)

The Fe_3_O_4_@*Sargassum* composite was prepared following the methodology of Díaz-Hernández et al. [22], substituting chitosan and genipin for *Sargassum* spp. and glutaraldehyde, respectively. Glutaraldehyde was used as a crosslinking agent between Fe_3_O_4_ nanoparticles and *Sargassum* spp. While EDTA was used as a functionalizing agent. 

Magnetic nanoparticles were synthesized by co-precipitation in the presence of solubilized algae extract, leading to its insertion within *Sargassum* spp. polysaccharides. A 2:1 molar mixture of FeCl_3_ and FeCl_2_ corresponding to 9.732 g and 4.342 g, respectively, was added to 30 mL of the solubilized algae and it was sonicated for 10 min using 70% amplitude (VC505, Sonics & Materials, Newtown, CT, USA). Then, the *Sargassum* spp. coated with magnetite was precipitated by adding a 4:1 solution of 20% p v^−1^ NaOH and 96% *v*/*v*^−1^ ethanol while stirring at 200 rpm and 25 °C for 12 h. The precipitate was washed in a 1:1 ratio with 50 mM phosphate buffer pH 7 and 96% *v*/*v*^−1^ ethanol until neutralization. The neutralized solids were dried in an oven at 80 °C for 5 h and grounded in a mortar until a fine powder was obtained.

The concentration of Fe_3_O_4_@*Sargassum*, glutaraldehyde, and EDTA on the adsorption of Hg^2+^ was assessed using a central composite design with three replicates. The minimum and maximum concentrations of magnetic composite, glutaraldehyde, and EDTA were 0.5–1.5% *w*/*w*^−1^, 0.2–1.0% *v*/*v*^−1^, and 0.05–1.0 g mL^−1^, respectively. The Fe_3_O_4_@*Sargassum* was suspended in the corresponding mixture of glutaraldehyde and EDTA in 50 mM phosphate buffer pH 4.5. The reaction mixture was vortexed every 10 min for 1 h at room temperature (20 ± 3 °C). The Fe_3_O_4_@*Sargassum*@EDTA was recovered with a 1.4 T permanent neodymium magnet and washed three times with a volume mixture of ethanol at 70% *v*/*v*^−1^—Milli-Q water (1:1) and dried at 80 °C. 

The solids yield (% p p^−1^) was determined as the ratio of final solids weight to initial solid weight multiplied by 100. The magnetic capacity of the synthesized composites was determined as the mass of the composites attracted by a permanent neodymium magnet (1.4 T); this was carried out by triplicate.

### 2.4. Determination of the Capacity of Adsorption of Hg^2+^ Using Fe_3_O_4_@Sargassum@EDTA

One hundred and fifty mg of Fe_3_O_4_@*Sargassum*@EDTA was incubated at pH 7 and 25 °C for 1 h with 50 mL of 100 mg Hg^2+^ L^−1^, prepared with HgCl_2_, and then 1.5 mL L^−1^ of HNO_3_, acid prevented evaporation of Hg^2+^. The mixture was centrifuged at 3000× *g* for 5 min, the supernatant was decanted, and the concentration of Hg^2+^ was quantified using the dithizone colorimetric method. The method forms an orange-colored dithizone-mercury complex, whose concentration is determined by absorbance at 490 nm [23]. The calibration curve was made from 0 to 0.8 mg of Hg^2+^ L^−1^.

The percentage of Hg^2+^ adsorption was obtained by multiplying by 100 the ratio of the difference between the final Hg^2+^ concentration and the initial Hg^2+^ concentration divided by the initial Hg^2+^ concentration.

### 2.5. Effect of pH, Time, and Temperature on the Fe_3_O_4_@Sargassum@EDTA Adsorption of Hg^2+^

The effect of pH and incubation time on Hg^2+^ adsorption was evaluated incubating at room temperature (~25 °C) and 200 rpm, with 150 mg of Fe_3_O_4_@*Sargassum*@EDTA with 50 mL of a 100 mg L^−1^ solution of Hg^2+^, the pH adjusted with concentrated HNO_3_ to 3, 5, and 7 values, and Hg^2+^ adsorption determined as previously described after 6, 12, and 24 h. The parameters that showed the highest adsorption of Hg^2+^ were then used to determine the effect of temperature (25, 30, 45, and 60 °C) on Hg^2+^ adsorption by Fe_3_O_4_@*Sargassum* @EDTA, as previously described.

### 2.6. Reusability of Fe_3_O_4_@Sargassum@EDTA

The reuse of the material after rinsing with 0.1 M NaOH, 0.1 M HCl, and 2 M EDTA was evaluated with Fe_3_O_4_@*Sargassum*@EDTA under the conditions that led to the highest adsorption capacity of Hg^2+^. Fifty mg of Fe_3_O_4_@*Sargassum*@EDTA was suspended in 10 mL of a 100 mg L^−1^ solution of Hg^2+^ and incubated for 12 h at 25 °C, pH 5, and 200 rpm, similar to Gai et al. [24]. Subsequently, the composite was removed from the reaction medium with an external permanent neodymium magnet (1.4 T), thoroughly rinsed three times, and constant stirring of 200 rpm for one hour, to eliminate free EDTA. Then, the rinsed composite was added to a fresh 100 mg L^−1^ solution of Hg^2+^ for subsequent reuse cycles.

### 2.7. Identification of Functional Groups, Thermal Property Analysis, Surface Roughness, and Magnetic Properties of the Composite

The Fourier transform infrared spectroscopy (FTIR) spectra were used to characterize the presence of functional groups in composites acquired from 16 scans on a Spectrum 100 infrared spectrometer (PerkinElmer, MA, USA) using 650–4000 cm^−1^. The thermal properties of the composites were analyzed in differential scanning calorimetry (DSC) using DSC822e (Mettler-Toledo, OH, USA) equipment with a heating rate of 10 °C min^−1^. High-purity indium was used as standard and dry nitrogen was used as the purge gas. The thermal curves were obtained in the range of −20 °C to 150 °C using the STARe Software (Mettler-Toledo, Greifensee, Switzerland).

The surface roughness of Fe_3_O_4_@*Sargassum* and Fe_3_O_4_@*Sargassum*@EDTA was observed through atomic force microscopy (AFM) analysis using an atomic force microscope (di Multimode V, Veeco, Plainview, NY, USA) in contact mode using silicon tips (Bruker RTESP Cantilever, Karlsruhe, Germany). The resonance frequency of scanning was 286–362 kHz with a spring constant of 20–80 N m^−1^, a scanning speed of 1 Hz, and a resolution of 256 × 256 pixels; the results were analyzed using Gwyddion Version 2.53 software (Okružní, Czech Republic). The quadratic roughness (*Rq*) of the samples was estimated by the square root of the deviation from an average plane of the peaks and surface valleys (Equation (2)):

(2) $$Rq=\sqrt{\frac{\Sigma Zi^{2}}{N}}$$

where *Rq* is the standard deviation of *Zi* values indicating roughness (nm); *Zi* is the difference in the height of *i* relative to the average height; and *N* is the number of points in the image.

The magnetization curve of Fe_3_O_4_@*Sargassum*@EDTA was measured at room temperature (~28 °C) with a maximum field of ±20 kOe using a Physical Property Measurement System (PPMS, Quantum Design, San Diego, CA, USA) magnetometer.

### 2.8. Statistical Analysis

All measurements were carried out in triplicate and the values were reported as mean ± standard deviation. An analysis of variance (ANOVA) to evaluate the central composite design and the Tukey test for mean comparison were conducted, respectively. Confirmatory experiments were made to evaluate the fit of the predicted model and optimize the empirical model. The standard error (S) and coefficient R^2^ were used to assess the significance of the model. The analysis was performed with Minitab 17.2.1 at a significance level of *p* < 0.05.

## 3. Results and Discussion

### 3.1. Solubilization of Sargassum *spp.* Polysaccharides

**Figure 1:**
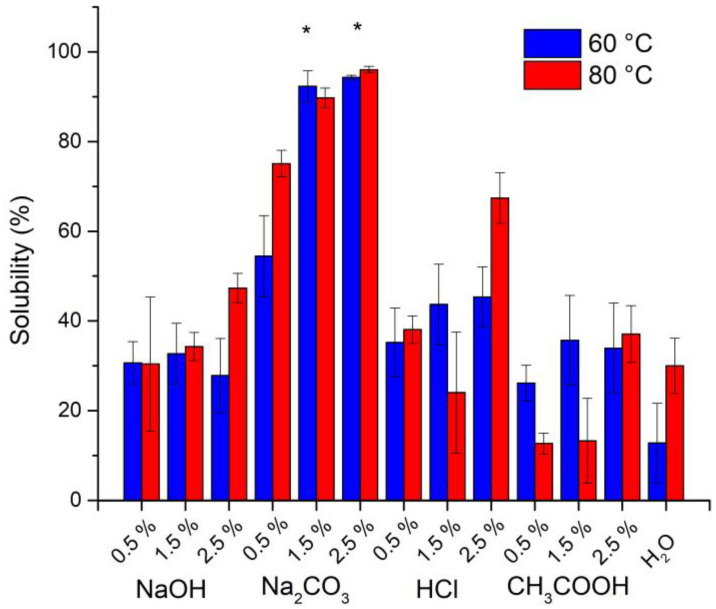
Solubilization of *Sargassum* sp. polysaccharides in solutions of NaOH, Na_2_CO_3_, HCl, and CH_3_COOH at 0.5, 1.5, and 2.5% p v^−1^ and heated to 60 (blue) and 80 °C (red). * Statistical difference in comparison of means by the Tukey test (significance at *p* < 0.05) (mean ± SD, n = 3).

The solubility of *Sargassum* spp. polysaccharides under acidic and basic conditions are shown in Figure 1. The maximum value of solubility was reached when using a 2.5% Na_2_CO_3_ solution at 60 °C and 80 °C with values of 96.3% and 94.3%, respectively. The lowest solubility was obtained with acetic acid at 80 °C and water at 60 °C.

Brown seaweed cell walls contain sulfated polysaccharides, i.e., laminarin, alginate, and fucoidan, with them having their own physical and chemical characteristics influenced by species, geographic location, season, and population age [17]. Moreover, the solubility of *Sargassum* spp. polysaccharides depends mainly on the nature and concentration of the solvent, due to their composition of polysaccharides [18]. Increased solubility observed with Na_2_CO_3_ agrees with procedures for alginate extraction [3,21]; however, here the absence of formaldehyde and HCl treatment yielded a brown product due to the presence of phenolic compounds.

Meanwhile, the increase in temperature slightly improved the percentage of *Sargassum* polysaccharide solubility, probably due to a decreased viscosity [25]. In addition, the use of hot water is not selective, as it not only removes water-soluble polysaccharides from algae such as fucoidan, alginate, and laminarin but also other water-soluble compounds from the seaweed [18]. Several studies reported *Sargassum* solubilization using temperatures of 80 °C for prolonged periods [3]; the production of carbonized materials requires temperatures up to 400 °C [9] and the preparation of other biosorbents such as sugarcane bagasse use acids and alkalis [1]. The use of Na_2_CO_3_ for *Sargassum* solubilization at 60 °C avoids the use of toxic and corrosive chemicals and reduces energy consumption resulting in an environmentally friendly technique for composite synthesis.

### 3.2. Synthesis of the Magnetic Sargassum Composite (Fe_3_O_4_@Sargassum) Functionalized with EDTA (Fe_3_O_4_@Sargassum@EDTA)

The magnetic composite of *Sargassum* spp. was synthesized by alkaline co-precipitation of iron nanoparticles immersed in the *Sargassum* extract. Subsequently, the Fe_3_O_4_@*Sargassum* was functionalized with EDTA. The predicted model for the effect of the concentration of glutaraldehyde, magnetite composite, and EDTA on the adsorption capacity of Hg^2+^ of Fe_3_O_4_@*Sargassum*@EDTA fits (R^2^ = 96.2%) to a second-degree polynomial equation (Equation (3)). The global solution conditions to maximize the adsorption capacity of Hg^2+^ were 1.3% magnetic composite, 1.0% glutaraldehyde, and 0.6 g of EDTA mL^−1^.

(3) $$Hg^{2+}\;adsorption=-117.7+77.2x_{1}-18.0x_{2}+322.4x_{3}+0.1x_{1}^{2}+97.0x_{2}^{2}-77.8x_{3}^{2}-6.3x_{1}x_{2}-140.1x_{1}x_{3}-170.7x_{2}x_{3}$$

where $x_{1}$ = % Fe_3_O_4_@*Sargassum*, $x_{2}$ = % glutaraldehyde, and $x_{3}$ = g of EDTA mL^−1^.

**Figure 2:**
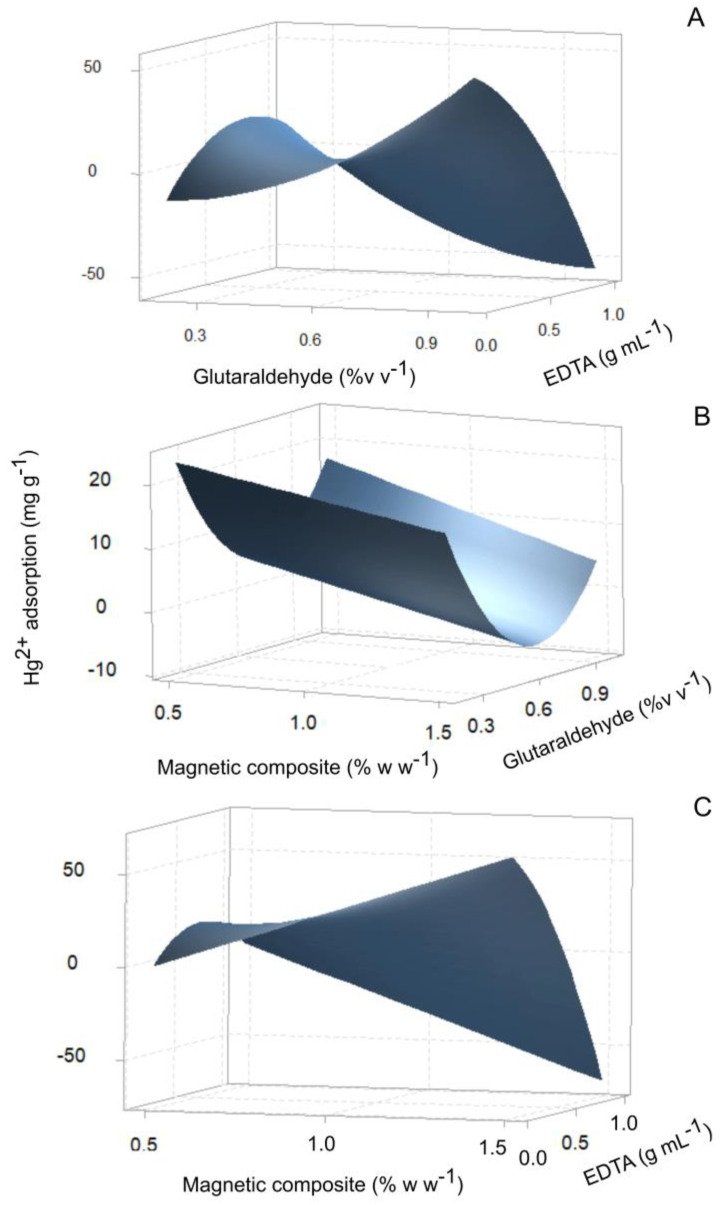
Response surface for the synthesis of the *Sargassum* composite as a function of (**A**) glutaraldehyde concentration (% *w*/*v*^−1^) and EDTA concentration (g mL^−1^), (**B**) the magnetic composite concentration (% *w*/*v*^−1^) crosslinked with glutaraldehyde (% *w*/*v*^−1^), and (**C**) the magnetic composite concentration (% *w*/*v*^−1^) functionalized with EDTA (g mL^−1^), to maximize the adsorption capacity of Hg^2+^.

The ANOVA is shown in Table 1. The concentration of glutaraldehyde and EDTA showed a significant linear effect on the adsorption capacity of Hg^2+^ of the magnetic compound. The quadratic variables, as well as the interactions, also showed a significant effect on the mercury removal capacity of the magnetic compound, leading to a curvature in the response surface as observed in Figure 2. 

The equation suggested that the concentration of EDTA generates the greatest positive impact on the Hg^2+^ adsorption capacity, and the greatest negative impact is due to the interaction between the glutaraldehyde and EDTA concentrations.

The solids yield in the *Sargassum* magnetic composite synthesis reaction and the percentage of attracted mass are shown in Table 2. The percentage of solids yield and attracted mass by a magnet of Fe_3_O_4_@*Sargassum*@EDTA were 60.1 ± 17.2% and 75.9 ± 6.6%; no significant difference with the magnetic composite before functionalization with EDTA was observed. The 1.21-fold decrease in the attracted mass by a magnet could be associated with the added non-magnetic EDTA on the surface of the magnetic material [19,20].

The use of a surface methodology analyzes the effects of the independent variables, generating a mathematical model that describes and optimizes a process with a reduced number of experimental trials [26]. The efforts to produce novel algae- and seaweed-based adsorbents using extraction, nanoparticles, and molecular and chemical modes have been reviewed regarding the metal binding capacity and the elucidation of inherent adsorption mechanisms [16]. Using biomass as heavy metal removal systems from effluents is frequently described as a low-cost, ecologically viable, and easy-to-run alternative [2]. Furthermore, brown algae *Sargassum* spp. extracts have proved to be excellent biosorbents for the removal of heavy metals in an aqueous solution [3,12]. On the other hand, some polysaccharides grafted with EDTA groups, spatially separated and opposite in charges, make adsorbents suitable for the simultaneous adsorption of multiple pollutants with different physicochemical properties, while also decreasing the EDTA–metal complex’s mobility [20]. Magnetic nanoparticles such as Fe_3_O_4_ are widely used for the preparation of composites [27], and the procedure shown in this work is fast and simple and requires no harmful chemicals as previously described [22]. In addition, magnetite nanoparticles have been used in Hg^2+^ adsorption processes due to their surface area and magnetic properties in water treatments [28]. The ability of efficient separation from effluent and the reuse of a magnetic adsorbent could provide several advantages for wastewater treatment. 

### 3.3. Effect of pH, Time, and Temperature on Fe_3_O_4_@Sargassum@EDTA Adsorption of Hg^2+^

**Figure 3:**
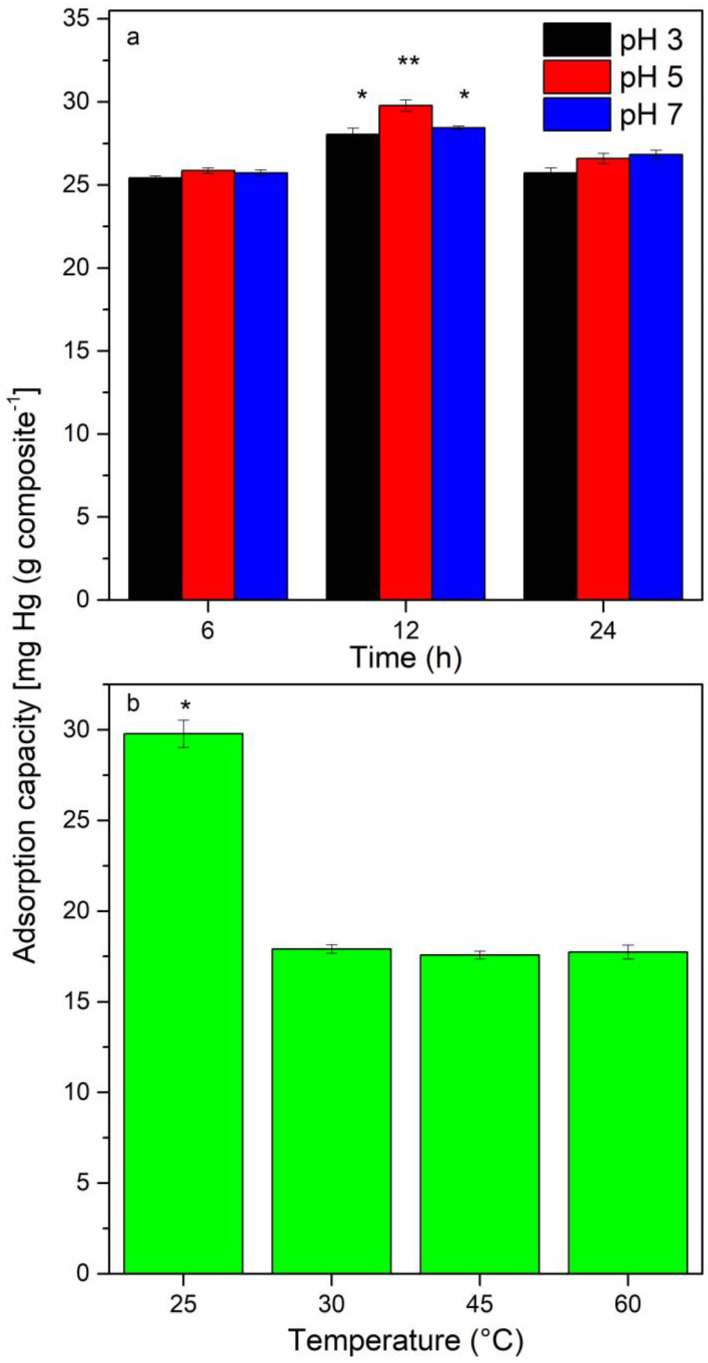
Adsorption capacity of mercury with the Fe_3_O_4_@*Sargassum*@EDTA (**a**) at pH 3 (black), 5 (red), and 7 (blue) for 24 h, 25 °C; and (**b**) at different temperatures for 12 h at pH 5 (mean ± SD, n = 3). * Statistical difference between treatment *p* > 0.05. ** Statistical difference between groups *p* > 0.05.

The adsorption capacity of mercury with the Fe_3_O_4_@*Sargassum*@EDTA at pH values of 3, 5, and 7 for 24 h and 25 °C is shown in Figure 3a. The adsorption capacity of mercury by the magnetic composite was pH and time dependent. A maximum adsorption capacity was obtained at 12 h (*p* < 0.05) in the pH values of 3, 5, and 7. By increasing the contact time from 6 to 12 h, the adsorption capacity increases 1.1-, 1.2-, and 1.1-fold for pH values of 3, 5, and 7, respectively; however, after 24 h of contact time, a desorption process was observed with decreases of 1.1-fold, respectively. Fe_3_O_4_@*Sargassum*@EDTA showed a continuous increase in adsorption capability after 24 h. At low pH, hydrogen and Hg ions compete for sorption sites, leading to electrostatic repulsion occuring among metal ions and protonated amino groups. As pH increases and the concentration of protons decreases, the competition for binding sites diminishes, and the sites such as amino and carboxylic groups of EDTA turn into dissociated forms exchanging protons with metallic ions in solution and forming chelates [1,29,30]. Furthermore, as the solution pH increased from 1 to 5, the electrostatic interaction between negatively charged COO^-^ groups of a chitosan composite adsorbent functionalized with EDTA and heavy metals dominates the adsorption process [20]. The composite hereby produced had a six-fold increase in the time necessary to reach equilibrium after adsorption [31]. 

The maximum adsorption at pH 6 was 50 times smaller (0.2 vs. 10 mg Hg^2+^ per mg adsorbent) and slower than that reported by Kumar et al. [32]; however, the synthesis method described here allows material recovery and reuse. Thus, its application would be advantageous in the final steps of water treatment where mercury concentrations are lower and acidic.

The effect of temperature on the adsorption capacity of mercury was analyzed at pH 5 as is shown in Figure 3b. A maximum adsorption capacity of 29.8 ± 0.75 mg of mercury per g was observed at 25 °C, while no significant difference effect (*p* < 0.05) was observed in the adsorption of mercury between temperatures 30, 40, and 60 °C. This temperature was implemented to evaluate mercury adsorption in subsequent experiments. A rise in temperature increases the diffusion rate of metal ions in water and the activity of functional groups conducting the adsorption of metal ions; however, in adsorption sites, deformation could occur at higher temperatures and limit the adsorption of metal ions [8]. The contact time influences the efficiency of the biosorbent; at the beginning of the process, the affinity for binding metal ions is high due to the availability of binding sites; as time passes, the affinity decreases until it reaches a saturation point as a result of having fewer available binding sites [2].

The maximum adsorption capacity was 29.8 ± 0.75 mg of mercury per g of the functionalized magnetic composite which was 1.3-fold higher than that obtained by Husein [4] with mandarin peel treated with NaOH at pH 5 and 25 °C but 1.2-fold lower than reported for Khoramzadeh et al. [1] using sugarcane bagasse at pH 4 and 30 °C. However, the sugarcane bagasse was used only once at pH 4 and had optimal adsorption at 50°C, leading to higher energetic costs. Carro et al. [29] found that the maximum capacity for mercury sorption (180 mg g^−1^) with a native and surface chemically modified *S. muticum* at pH 5 was reached after 5 h. Mohammed et al. [12] studied the kinetic data of calcium alginate thin films derived from *S. natans* for the selective adsorption of heavy metals for up to 6 h. On the other hand, Husein [4] and Khoramzadeh et al. [1] found a negligible effect of temperature on mercury biosorption at equilibrium using raw and chemically modified Egyptian mandarin peel and sugarcane bagasse, respectively.

### 3.4. Reusability of Fe_3_O_4_@Sargassum@EDTA

**Figure 4:**
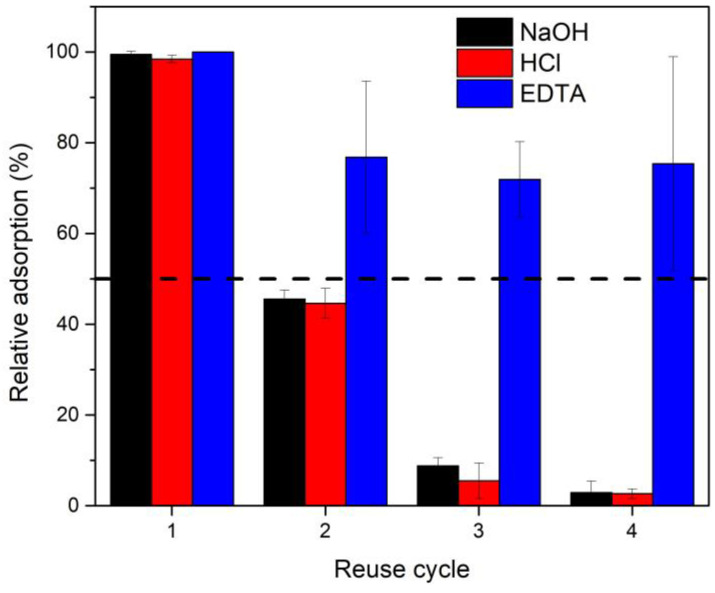
Consecutive cycles of Hg^2+^ adsorption of the magnetic composite after 12 h at 25 °C, pH 5, and 200 rpm. Amounts of 0.1 M NaOH (black), 0.1 M HCl (red), and 2 M EDTA (blue) were used to rinse the magnetic composite in each cycle. Each reaction cycle lasted 12 h and the adsorption capacity in the first cycle was set as 100 % (mean ± SD, n = 3).

The regeneration and stability of an adsorbent are of great significance for its practical application, besides the adsorption capability. The consecutive cycles of Hg^2+^ adsorption using the magnetic composite are shown in Figure 4. Mercury adsorption in the first cycle achieved 100% removal in contrast to a 95% removal rate reported with activated carbon obtained from coconut buttons at pH 7 by [9]. The rinsing solution showed an effect on the adsorption capacity over consecutive reuse cycles. The relative adsorption decreased by 25% from the second to the fourth reuse cycle after rinsing with 2 M EDTA while a decrease of 55%, 94%, and 97% was observed in the second, third, and fourth cycles, respectively, by rinsing the composite with 0.1 M NaOH and 0.1 M HCl. The loss of adsorption efficiency increasing the reuse cycles could be due to the loss or damage of biomass and the possibility of acid/basic deactivation of binding sites [18]. In addition, the magnetic composite was successfully removed from the reaction medium with an external permanent neodymium magnet (1.4 T) in each cycle.

Table 3 shows that the former study used an initial Hg^2+^ concentration above the other studies, similar to those used with carbonized mandarin peel [4]. Despite the material achieving maximum adsorption in a shorter time, the energy for producing the material is high and was not evaluated for reuse cycles. The study of pure and modified activated carbon [24] used an initial mercury concentration that was one thousand times lower than the concentration in our study. However, the amount of adsorbent used in that study was 38 times lower than the one used here. Native and modified *Sargassum muticum* achieved almost 50% Hg^2+^ removal using only half the amount of adsorbent and initial concentration compared to our study. However, the contact time for adsorption was four times longer, and the study evaluated only one cycle [29]. Similar results were obtained with functionalized magnetite; however, the contact time was nearly four times shorter. Additionally, the desorbing agents used in that study were more corrosive than EDTA [28]. Finally, Khoramzadeh et al. [1] found that sugar bagasse and its modified materials achieved the maximum Hg^2+^ adsorption in a shorter time than in our study, using an initial concentration that was nearly 1.4 times lower.

Regeneration without damaging the capacity of the biosorbent is crucial for bisorbent technology development; and a successful desorption process requires the proper selection of eluents, which strongly depends on the type of biosorbent and must be no damaging to the biomass, less costly, environmentally friendly, and effective [33]. Some studies refer to alkalis as effective desorbing agents for desorbing heavy metals from chemically modified adsorbents. At the same time, acids are favorable for the desorption processes of bio-adsorbents, where their protons compete with metals for the active surface sites, especially, the chelating agent, EDTA [16,32]. Kumar et al. [32] also reported EDTA as the best elution agent leading to higher mercury removal efficiency, followed by acidic solutions and NaOH; similar relative adsorptions were reported after four cycles.

Awual [34] reported a mesoporous composite with the ability to adsorb Hg^2+^ with 89% efficiency after eight continuous cycles, while Chen et al. [20] reported initial adsorptions of Cu^2+^ and Pb^2+^ by Chitosan@Fe_3_O_4_@EDTA of 180 and 140 mg g^−1^, respectively, dropping to 100 and 150, respectively, after eight reuse cycles. After the biosorption process loses its efficiency, metal-contaminated biomass should be incinerated to avoid environmental damage and the possibility of metal recovery [14]. Gai et al. [24] reported less than 80% adsorption with activated carbon and organoclay while using an initial Hg^2+^ concentration 1000 times smaller than the concentration in the present work by saturating the pores aided with vacuum; here, we did not implement such a process. Mesoporous systems have a high affinity for metal recovery; however, they are expensive [24] while low-cost alternatives such as activated carbon have high energy requirements; therefore, replacing them with waste-based materials is advantageous. Hence, the *Sargassum* magnetic composite is a promising alternative biosorbent for wastewater treatment.

The concentration evaluated in this work was 100 mg L^−1^, while worldwide wastewater effluents have concentrations around 0.01–2.5 mg L^−1^ [7] or even fewer than 8 ng L^−1^ [35], therefore showing a major Hg^2+^ adsorption capacity. Suess et al. [35] also observed that the Hg^2+^ removal in wastewater treatment plants is diminished as the initial concentration in the effluent increases, evaluating a maximum concentration of 59 ng L^−1^; thus, the adsorption achieved with Fe_3_O_4_@*Sargassum*@EDTA is notable. Accordingly, this composite has the potential for Hg^2+^ removal as a polishing technology for wastewater. In addition, the thorough wash of the composite between cycles could minimize the EDTA release to the effluent.

### 3.5. Identification of Functional Groups, Thermal Property Analysis, the Surface Roughness of the Composite, and its Magnetic Properties

**Figure 5:**
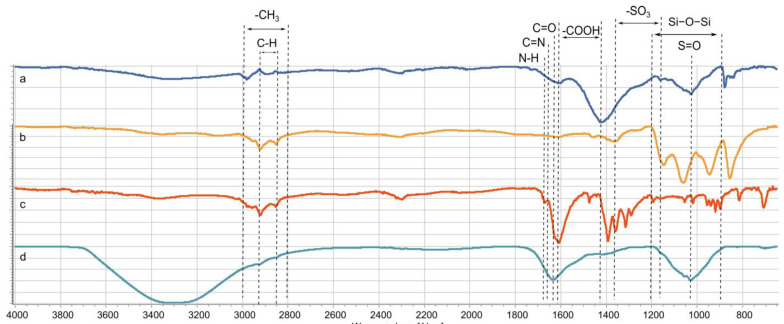
FTIR spectra of (**a**) *Sargassum* spp., (**b**) Fe_3_O_4_@*Sargassum*, (**c**) Fe_3_O_4_@*Sargassum*@EDTA, and (**d**) Fe_3_O_4_@*Sargassum*@EDTA≡Hg^2+^.

The presence of functional groups in the composite materials that can interact with the Hg^2+^ ion was confirmed by Fourier transform infrared spectroscopy (FTIR) tests. The spectra obtained by FTIR for the *Sargassum*, Fe_3_O_4_@*Sargassum*, Fe_3_O_4_@*Sargassum*@EDTA, and Fe_3_O_4_@*Sargassum* @EDTA after mercury adsorption can be seen in Figure 5. *Sargassum* showed absorption bands at 2852 and 2920 cm^−1^ indicating a stretching of the C–H bond [18]. The bands around 1419 cm^−1^ and 1604 cm^−1^ are characteristic of -COOH carboxyl groups [31]. There was a displacement of the 1419 cm^−1^ band at 1393 cm^−1^ and 1407 cm^−1^ in the Fe_3_O_4_@*Sargassum* and Fe_3_O_4_@*Sargassum* @EDTA samples, respectively, while for the second carboxyl group, there was a displacement at 1607 and 1629 cm^−1^ in the Fe_3_O_4_@*Sargassum*@EDTA and Fe_3_O_4_@*Sargassum*@EDTA≡Hg^2+^ samples, respectively. The carboxyl groups could be involved in crosslinking reactions and the chelating effect of EDTA on mercury. The band at 1630 cm^−1^ characteristic of the C=O carbonyl group present in EDTA [36] was observed in the samples Fe_3_O_4_@*Sargassum*@EDTA and Fe_3_O_4_@*Sargassum*@EDTA≡Hg^2+^. The adsorption band at 1030 cm^−1^ indicated the presence of the S=O bond in *Sargassum* spp. [31] a characteristic component of fucoidans [2], shifting to 1055 in Fe_3_O_4_@*Sargassum*, Fe_3_O_4_@*Sargassum*@EDTA, and Fe_3_O_4_@*Sargassum*@EDTA≡Hg^2+^, which indicates the structural modifications suffered by *Sargassum* because of the insertion of magnetic material, crosslinking, and mercury chelation. In the Fe_3_O_4_@*Sargassum*@EDTA and Fe_3_O_4_@*Sargassum*@EDTA≡Hg^2+^ samples, the bands observed at 1655 and 1668 cm^−1^ are characteristic of the C=N bond indicating the crosslinking between the amino group of sargassum extract and glutaraldehyde [20]. In addition, the displacement of the bands characteristic of the –NH_2_, -COOH, and -C=O functional groups after the crosslinking process indicates the formation of covalent bonds [22]. Fe_3_O_4_@*Sargassum*@EDTA showed a band at 1670 cm^−1^ characteristic of the N–H bond vibration corresponding to amides. All samples showed absorption bands between 3000 and 2800 cm^−1^, characteristic of the methylene group. Likewise, the bands between 1200 and 900 cm^−1^ are related to the overlapping and complexation of polysaccharides and siloxane [15]. Finally, those bands between 1361 and 1160 cm^−1^, observed in all samples but with greater intensity in Fe_3_O_4_@*Sargassum*@EDTA, correspond to sulfone groups (S=O) while the bands between 700 and 1000 cm^−1^ correspond to S-O vibrations [37]. The presence of -COOH, -C=O, and -NH_2_ groups enabled the biosorption of mercury from Fe_3_O_4_@*Sargassum*@EDTA [2].

The pseudo-second-order model for adsorption isotherm has been reported to predict the sorption mechanism of divalent metal ions in algal biomasses due to a chemisorption process where the covalent bonding between the carboxyl groups creates sites for metal ion sharing or exchange [12,20,32].

**Figure 6:**
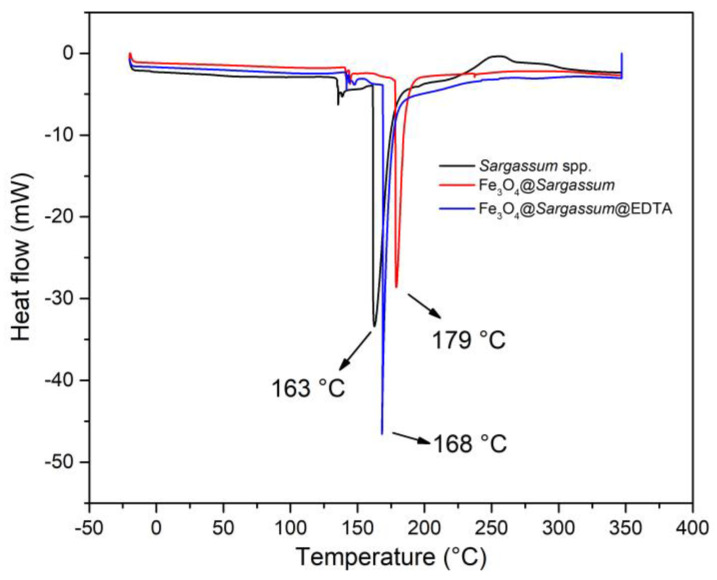
Adsorption DSC measurement curves of *Sargassum* spp. (black), Fe_3_O_4_@*Sargassum* (red), and Fe_3_O_4_@*Sargassum*@EDTA (blue).

DSC measurement curves to characterize the phase transition of *Sargassum* spp., Fe_3_O_4_@*Sargassum*, and Fe_3_O_4_@*Sargassum*@EDTA due to the temperature and heat flow are shown in Figure 6. A narrow endothermic peak in *Sargassum* spp., Fe_3_O_4_@*Sargassum*, and Fe_3_O_4_@*Sargassum*@EDTA at 163 °C, 179 °C, and 168 °C was observed, respectively. Besides this major endothermic peak, there is a small signal of any other phase changes in the DSC curve which could indicate other macromolecules such as proteins extracted along with the polysaccharides contained in the cell wall structure of *Sargassum* spp. [38].

The phase transition temperature of sargassum was lower than the composites and could be due to inter- and intramolecular hydrogen bonding formed by the polysaccharide hydroxyl and carboxylate groups from sodium alginate [39]. In addition, the difference in the thermal events may be explained by the crosslinking degree and the functionalization with Fe_3_O_4_ and EDTA. 

**Figure 7:**
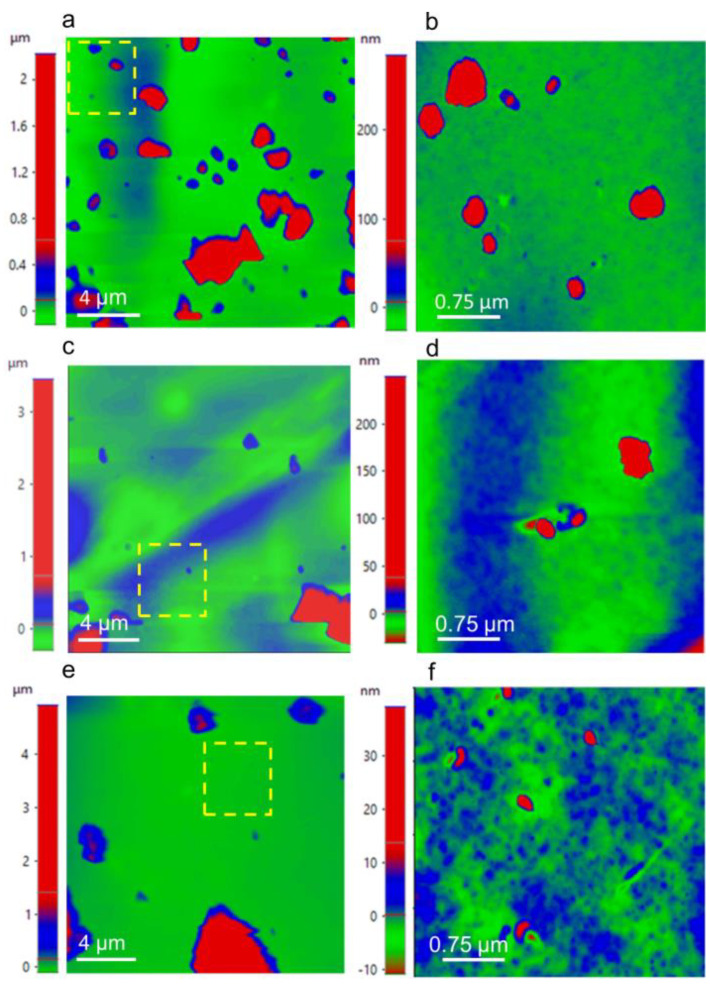
Atomic force microscopy (AFM) micrographs of Fe_3_O_4_@*Sargassum* (**a**,**b**), Fe_3_O_4_@*Sargassum*@EDTA (**c**,**d**), and Fe_3_O_4_@*Sargassum*@EDTA≡Hg^2+^ (**d**,**e**). The delimited area in panels (**a**,**c**,**e**) (dashed lines) corresponds to the magnification zone of panels (**b**,**d**,**f**).

The AFM micrographs evidenced the change in the surface roughness of the composites in Figure 7. The surface roughness of Fe_3_O_4_@*Sargassum*, Fe_3_O_4_@*Sargassum*@EDTA, and Fe_3_O_4_@*Sargassum*@EDTA≡Hg^2+^ was 157.3 ± 145.6 nm, 125.2 ± 196.9 nm, and 281.0 ± 400.0 nm, respectively. As shown in the micrographs, the magnetic composite’s surface roughness was more homogeneous than the functionalized composite before and after the Hg^2+^ adsorption. The micrographs in Figure 7 show an increasing roughness of the magnetic composite (Figure 7a,b) after crosslinking with EDTA (Figure 7c,d) and then after Hg^2+^ adsorption (Figure 7e,f). The latter shows a direct correlation between composite and surface complexity leading to the formation of porous structures (roughness) in the magnetic composite [20]. The roughness of both the functionalized and Hg^2+^ adsorbed composites was similar (Figure 7c,d,e,f) showing smooth surfaces and dispersed high valleys. Some crosslinked composites have shown irregular and undefined shapes where agglomerates can be seen, contrasting with the homogeneity of non-functionalized composites [22]. The interaction of surface functional groups of the composite along adsorbed Hg^2+^ may form agglomerates, thus enhancing the surface roughness. In addition, the co-precipitation method for synthesis of Fe_3_O_4_ nanoparticles used in this study has shown signals corresponding to the planes characteristic of Fe_3_O_4_ and regular, defined, and polyhedral shapes, such as tetra-, hexa-, and octahedrons [22,40].

**Figure 8:**
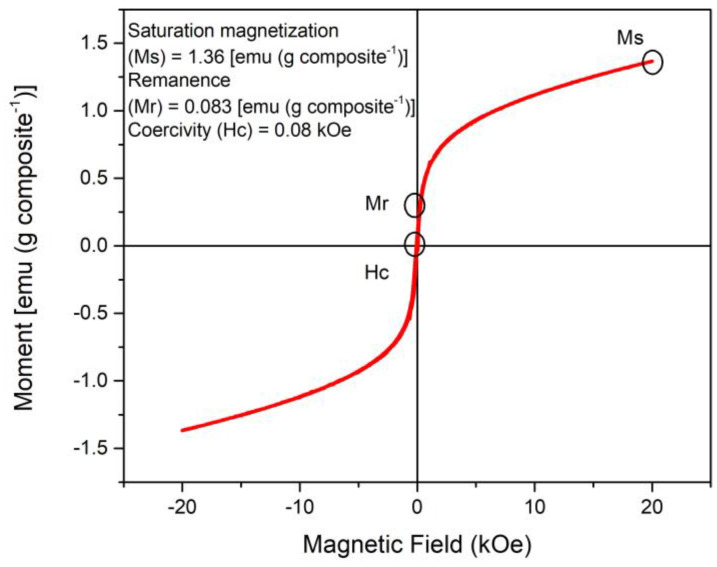
Magnetization curve of the magnetic composite Fe_3_O_4_@*Sargassum*@EDTA at ~28 °C.

The development of magnetization (moment) depending on the magnetic field at ~28 °C is shown in Figure 8. The saturation magnetization (Ms) of the Fe_3_O_4_@*Sargassum*@EDTA composite was calculated by linear extrapolation as 1.4 [emu (g composite^−1^)]. The magnetic hysteresis loop of Fe_3_O_4_@*Sargassum*@EDTA showed ferromagnetic properties allowing fast solid/liquid separation and recovering the magnetic composite from an aqueous mixture through a neodymium magnet. The magnetic remanence (Mr = 0.08 [emu (g composite^−1^)]) and coercivity (Hc = 0.08 kOe) were practically zero; thus suggesting the Fe_3_O_4_@*Sargassum*@EDTA did not retain magnetization and is a magnetically soft material.

The crosslinking within Fe_3_O_4_@*Sargassum*@EDTA led to decreased magnetic properties in comparison to previously reported Fe_3_O_4_. This decrease has been observed in several magnetite nanocomposites and could be attributed to the added non-magnetic mass to the composites [19,20,22].

## 4. Conclusions

Within the synthesis of the magnetic composite, the Hg^2+^ adsorption capacity of the Fe_3_O_4_@*Sargassum*@EDTA composite is mainly determined by the concentration of EDTA and the interaction between it and glutaraldehyde. The Hg^2+^ adsorption capacity of the synthesized composite is affected by temperature, pH, and reaction time. The factor with the major effect on the adsorption capacity is the pH, as well as the presence of hydroxy, carboxy, amino, and sulfonate groups. The composite showed a limited capacity for reuse after rinsing with EDTA. It was possible to transform a harmful residue, such as *Sargassum* spp., into a Fe_3_O_4_@*Sargassum*@EDTA compound with Hg^2+^ adsorption capacity that can magnetically be recovered, generating a potential biosorbent for industrial wastewater treatment. Further investigations comparing the life cycle assessment, technical-economic, and energetic analysis of common adsorbents versus sargassum extract will provide enough data for the industrial-scale level. In addition, in our follow-up studies, we will further characterize the structure and morphology of the composite using techniques such as dispersive X-ray, scanning electron, and transmission electron microscopy. Additionally, we will optimize the adsorbent for natural wastewater treatment, considering factors such as time, dose, concentration, and pH.

## Figures and Tables

**Table 1:** Analysis of variance (ANOVA) of the response surface methodology for the synthesis of the *Sargassum* magnetic composite with the composite, crosslinked with glutaraldehyde, and functionalized with EDTA, maximizing the adsorption capacity of Hg^2+^.

Source	DF	Adjusted Sum of Squares	Adjusted Mean Squares	F Value	*p*-Value Prob > F
Model	9	157.166	17.4629	16.88	0.001
Lineal	3	23.246	7.7486	7.49	0.019
Magnetic composite (%) *w*/*v*^−1^	1	4.629	4.6295	4.47	0.079
Glutaraldehyde (%) *w*/*v*^−1^	1	19.351	19.351	18.71	0.005
EDTA (g mL^−1^)	1	19.388	19.3879	18.74	0.005
Square	3	114.931	38.3105	37.03	0
Magnetic composite (%) *w*/*v*^−1^	1	82.301	82.3007	79.55	0
Glutaraldehyde (%) *w*/*v*^−1^	1	17.554	17.5535	16.97	0.006
EDTA (g mL^−1^)	1	17.734	17.7336	17.14	0.006
Interaction	3	21.641	7.2137	6.97	0.022
$\mathrm{Magnetic}\;\mathrm{composite}\;(\%)\;w/v^{-1}\;\times$ Glutaraldehyde (%) *w*/*v*^−1^	1	18.532	18.5324	17.91	0.005
$\mathrm{Magnetic}\;\mathrm{composite}\;(\%)\;w/v^{-1}\;\times$ EDTA (g mL^−1^)	1	18.841	18.8414	18.21	0.005
$\mathrm{Glutaraldehyde}\;(\%)\;w/v^{-1}\;\times$ EDTA (g mL^−1^)	1	18.826	18.8256	18.2	0.005
Error	6	6.207	1.0345		
Lack of fit	1	0.173	0.1727	0.14	0.721
Pure error	5	6.035	1.2069		
Total	15	163.373			

R^2^: 0.96, DF: degree of freedom, significance level: *p* < 0.05.

**Table 2:** The solids yield and attracted mass by a permanent neodymium magnet (1.4 T) of the magnetic composite with and without EDTA (mean ± SD, n = 3).

Composite	Solids Yield (%)	Attracted Mass by a Magnet (%)
Fe_3_O_4_@*Sargassum*	86.0 ± 7.20 ^a^	92.1 ± 3.40 ^a^
Fe_3_O_4_@*Sargassum*@EDTA	60.1 ± 17.2 ^a^	76.0 ± 6.62 ^b^

The superscript letter in the data indicate the statistical difference by the Tukey test, *p* < 0.05.

**Table 3:** Comparison of Hg^2+^ removal using different adsorbent materials.

Adsorbents	Initial Hg^2+^ Concentration (mg L^−1^)	Final Hg ^2+^ Concentration (mg L^−1^)	Amount of Adsorbent Used (g L^−1^)	Contact Time for Maximum Adsorption (min)	q_e_ = Amount of Hg^2+^ Adsorbed per Adsorbent (mg/g)	Number of Reuse Cycles	Desorbing Agent	Optimal Temperature (°C)	Reference
*Sargassum*@magnetite composite EDTA-functionalized	100	0 (cycle 1), 23 (cycle 2), 28 (cycle 3), 25 (cycle 4)	5.0	720	29.8	4	0.1 M NaOH, 0.1 M HCl, 2 M EDTA	25	Former study
Sugarcane bagasse native, NaOH-treated, and HCl-treated	76	5.0, 8.0, and 13	5.0	5, 45, and 15	35.7	Single use	Not used	50	[1]
Calcium-alginate from *Sargassum* sp.	Ni^2+^ 92–99, Cu^2+^ 99–108	41, 82	1.0	180, 360	Not reported	Single use	Not used	50	[3]
Egyptian mandarin peel dried, NaOH treated, andcarbonized	100	Not reported	5.0	60	10, 13, and 17	Single use	Not used	20	[4]
Activated carbon, sulfur-impregnated activated carbon, and andorganoclay	0.1	0.055, 0.072, and 0.076	0.13	240	0, 0, and 0.4	Single use	Not used	Not reported	[24]
Schiff-base functionalized magnetic Fe_3_O_4_ prepared by the one pot subsequent homogeneus method andtraditional heterogeneus method	401	Not reported	1.0	180	0.57, 0.52	5.0	5% thiourea-0.5 mol L^−1^ nitric acid	35	[28]
*Sargassum muticum* native and treated for modification of carboxyl groups	501	401, 326	2.5	2880	0.9, 0.85	Single use	Not used	25	[29]

Note: The values presented were calculated by the authors from the data presented and are not exact.

## Data Availability

The data presented in this study are available from the corresponding author upon request.
